# Valve-in-valve transcatheter aortic valve replacement for bioprosthetic valve failure complicated by hypo-attenuated leaflet thickening: a case report

**DOI:** 10.1093/ehjcr/ytag032

**Published:** 2026-01-27

**Authors:** Kuang-Chien Chiang, Kang Liu, Wen-Jeng Lee, Mao-Shin Lin, Li-Tan Yang

**Affiliations:** School of Medicine, National Taiwan University, No. 1, Sec. 1, Ren'ai Rd., Zhongzheng Dist., Taipei 100233, Taiwan; School of Medicine, National Taiwan University, No. 1, Sec. 1, Ren'ai Rd., Zhongzheng Dist., Taipei 100233, Taiwan; Department of Medical Imaging, National Taiwan University Hospital, No. 7, Zhongshan S. Rd., Zhongzheng Dist., Taipei 100225, Taiwan; Department of Internal Medicine and Cardiovascular Center, National Taiwan University Hospital, No. 7, Zhongshan S. Rd., Zhongzheng Dist., Taipei 100225, Taiwan; Department of Internal Medicine and Cardiovascular Center, National Taiwan University Hospital, No. 7, Zhongshan S. Rd., Zhongzheng Dist., Taipei 100225, Taiwan

**Keywords:** Aortic valve, Valve replacement, Stenosis, Thrombosis, Echocardiography, Computed tomography, Case report

## Abstract

**Background:**

Bioprosthetic valve dysfunction (BVD) is a common complication after aortic valve replacement. Valve-in-valve transcatheter aortic valve replacement (ViV-TAVR) offers a less invasive alternative to redo surgery. However, research on hypo-attenuated leaflet thickening (HALT) following ViV-TAVR remains limited.

**Case summary:**

A 74-year-old man with hypertension, transient ischaemic attack, and prior aortic and mitral bioprosthetic valve replacements for infective endocarditis 10 years ago demonstrated elevated transaortic pressure gradients and severely reduced effective orifice area index without paravalvular leakage on transthoracic echocardiography (TTE) despite initial asymptomatic status. Serial TTE monitoring every 6 months initially suggested patient-prosthesis mismatch, later progressing to structural valve degeneration. However, transoesophageal echocardiography revealed no evident leaflet thickening or limited aortic valve opening. Furthermore, computed tomography (CT) confirmed proper aortic valve opening and further excluded pannus and thrombus. Due to symptom progression, worsening echocardiographic haemodynamics, and elevated NT-proBNP, ViV-TAVR with pre-procedural balloon valve fracture was performed, resulting in improved haemodynamics at 1-month follow-up. Post-procedural follow-up CT at 5 months revealed HALT, accompanied by only a mild elevation in peak aortic flow velocity on transthoracic echocardiography. Warfarin at a dose of 1 mg daily was initiated, resulting in thrombus resolution on 6-month follow-up CT, after which therapy was continued with regular TTE monitoring.

**Discussion:**

This case highlights the importance of multimodality and serial imaging in diagnosing and managing complex BVD. The observed HALT resolution raises questions about anticoagulation strategies after ViV-TAVR, especially in the absence of definitive guidelines.

Learning points

- To acknowledge the importance of multimodality and serial follow-up imaging in differentiating patient-prosthesis mismatch from leaflet thrombosis and pannus formation.
- Hypo-attenuated leaflet thrombosis after valve-in-valve TAVR can resolve with anticoagulants, though the optimal agent and its long-term prognostic impact warrant further investigation.

## Introduction

Differential diagnosis and management of bioprosthetic valve dysfunction (BVD) are critical after aortic valve replacement (AVR).^1,2^ While valve-in-valve transcatheter AVR (ViV-TAVR) offers a less invasive alternative to surgical re-intervention, research on hypo-attenuated leaflet thickening (HALT) following ViV-TAVR remains limited.^3^ We present a complex case involving progressive structural and non-structural BVD, the development of HALT after ViV-TAVR, and its resolution following warfarin therapy.

## Summary figure

**Figure:**
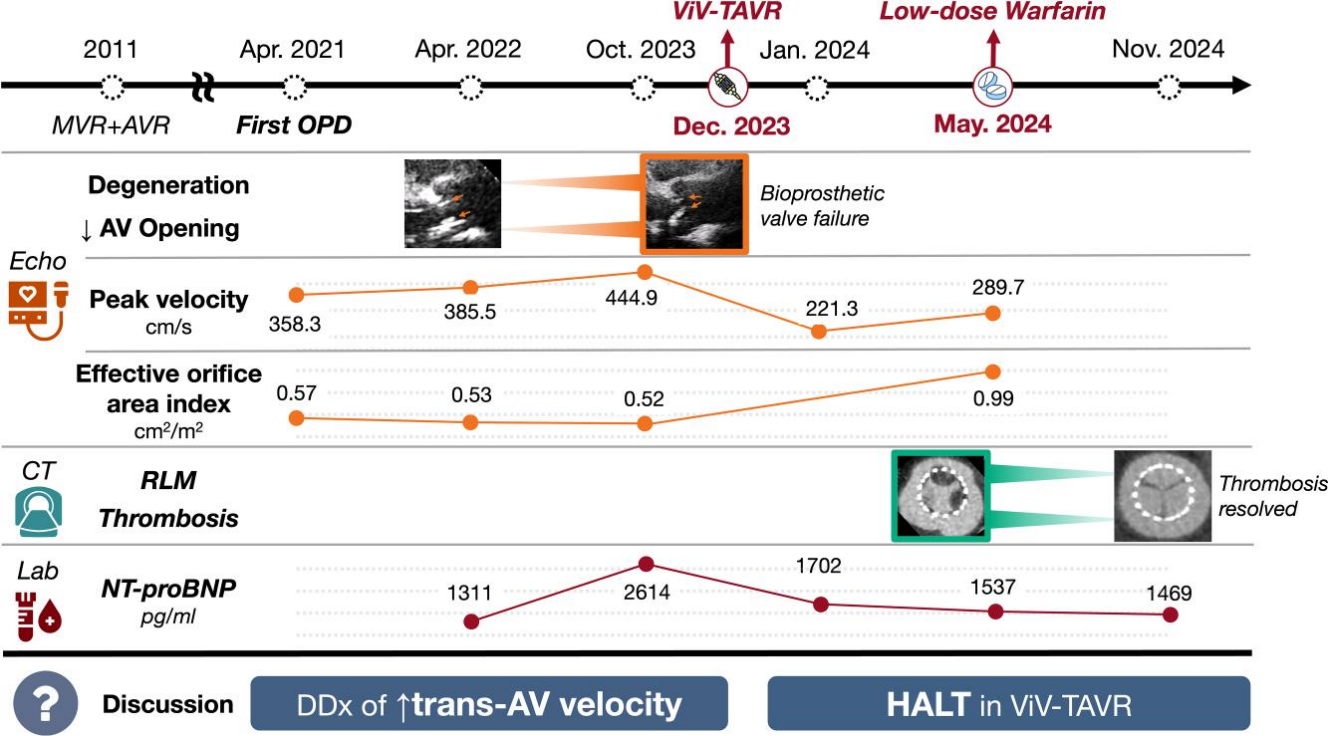
CT, computed tomography; Echo, echocardiography; HALT, hypo-attenuated leaflet thickening; MVR, mitral valve replacement; NT-proBNP, N-terminal pro-B-type natriuretic peptide; OPD, outpatient department; RLM, restricted leaflet motion; *trans*-AV, transaortic valve; ViV-TAVR, valve-in-valve transcatheter aortic valve replacement.

## Case presentation

A 74-year-old male with prior aortic (21 mm Carpentier-Edwards PERIMOUNT Magna Ease) and mitral valve replacements for infective endocarditis 10 years ago presented to our clinic in 2021 for follow-up of bioprosthetic valve function. Although asymptomatic (New York Heart Association Functional Class I), physical examination revealed a grade II/VI early systolic murmur at the left upper sternal border. Past medical history included hypertension and transient ischaemic attack.

**Figure 1:**
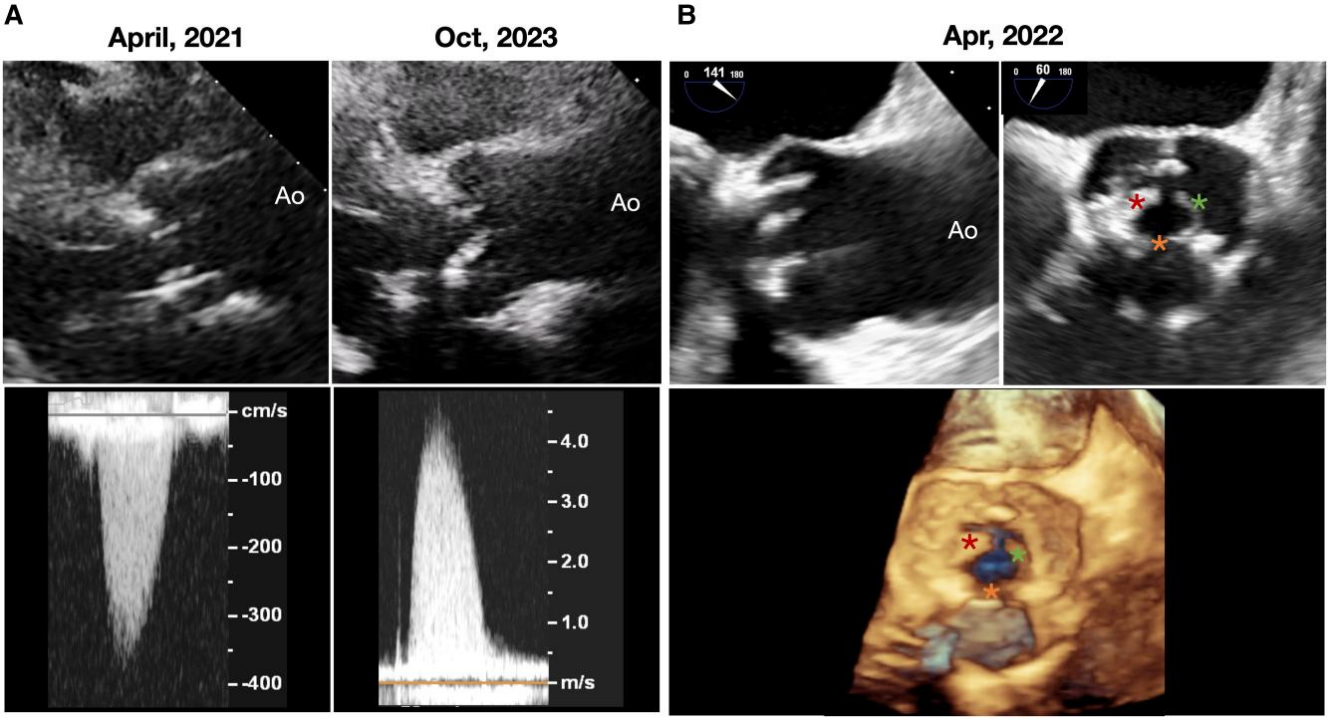
Echocardiographic assessment of bioprosthetic valve dysfunction. (*A*) Transthoracic echocardiography 2D imaging (top) and continuous-wave Doppler (bottom) showing valve morphology, mobility, and transvalvular gradient. (*B*) Transoesophageal echocardiography 2D (top) and 3D (bottom) images showing leaflet morphology and motion.

Initial transthoracic echocardiography (TTE) revealed elevated transaortic valve pressure gradient [peak velocity (pVel): 358.3 cm/s, mean pressure gradient (MPG): 29.5 mmHg, acceleration time: 80 ms, dimensionless velocity index: 24.8%; *Figure 1A*, left] and severely reduced effective orifice area index (EOAi, 0.57 cm^2^/m^2^) without paravalvular leakage. Due to relatively normal valve morphology, motion, and absence of leaflet calcification (see Supplementary material online, *Video S1*), valvular degeneration was considered less likely. The differential diagnosis included patient-prosthesis mismatch (PPM), pannus formation, and valve thrombosis. However, the lack of baseline postoperative TTE made it challenging to distinguish PPM from other causes.

**Figure 2:**
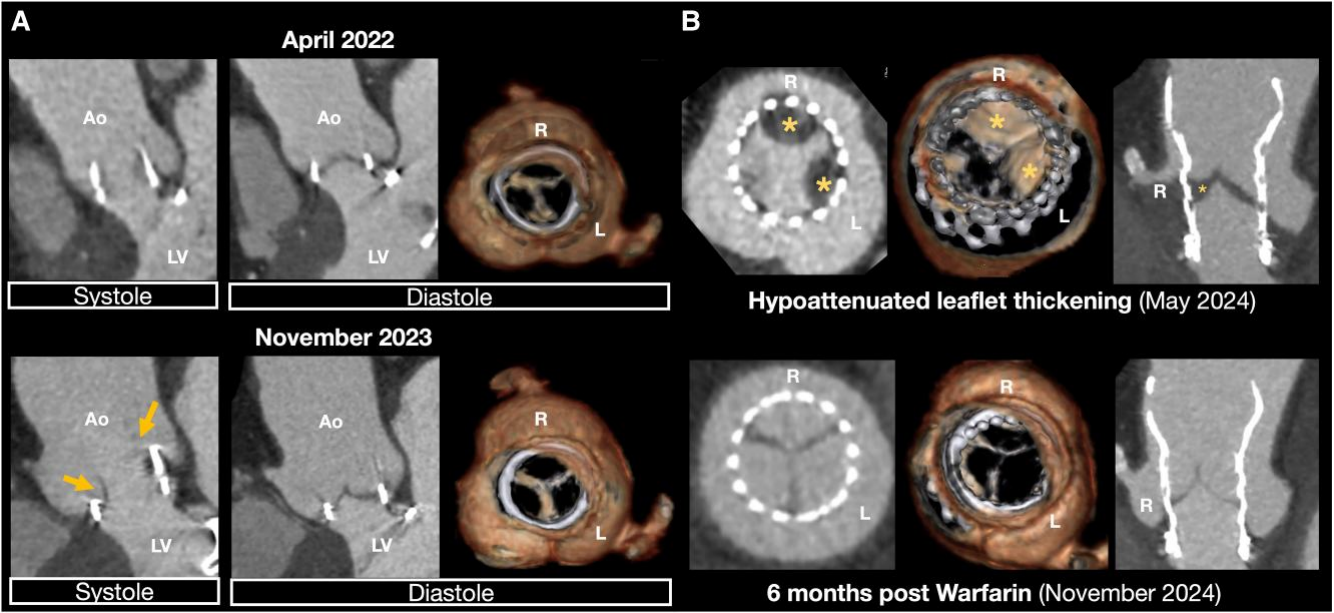
Electrocardiogram-gated cardiac computed tomography of the aortic valve. (*A*) Initial computed tomography in 2022 and computed tomography for pre-procedural planning of valve-in-valve transaortic valve replacement in 2023. The arrowhead indicates reduced leaflet motion during the systolic phase. (*B*) Post-procedural follow-ups showing hypo-attenuated leaflet thickening (asterisks) and resolution after warfarin therapy.

As the patient remained asymptomatic, conservative management of serial TTE monitoring every 6 months was pursued. In April 2022, TTE showed pVel progression (385.5 cm/s), accompanied by elevated NT-proBNP (1311 pg/mL). However, transoesophageal echocardiography revealed no evident leaflet thickening or limited aortic valve opening (*Figure 1B*). Computed tomography (CT) confirmed proper aortic valve opening without evidence of thrombus or pannus (*Figure 2A*, top).

By October 2023, the patient developed progressive exertional dyspnoea, with NT-proBNP rising significantly within 6 months (April 2023:1680 pg/mL; October 2023:2614 pg/mL). Transthoracic echocardiography suggested bioprosthetic valve degeneration (pVel: 444.9 cm/s, MPG: 41.8 mmHg, EOAi: 0.52 cm^2^/m^2^), evidenced by limited aortic valve opening (*Figure 1A*, right; Supplementary material online, *Video S2*), with preserved left ventricular ejection fraction and reduced left ventricular global longitudinal strain (LVGLS: 14%).

**Figure 3:**
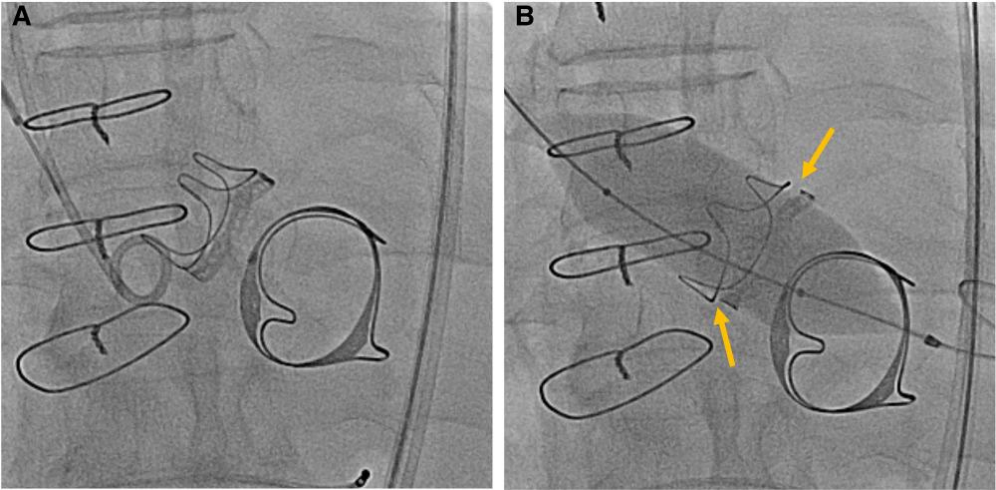
Valve-in-valve transcatheter aortic valve replacement with balloon valve fracture before valve implantation. (*A*) Pre-balloon valve fracture imaging of the original bioprosthetic valve. (*B*) Deformed and fractured valve frame after balloon valve fracture.

Given the patient’s symptomatic decline, worsening echocardiographic haemodynamics, and complex surgical history—including frontotemporal craniotomy for subdural haemorrhage in June 2023 and prior open-heart surgery—ViV-TAVR was chosen over redo surgical AVR (Society of Thoracic Surgeons score:2.97%; European System for Cardiac Operative Risk Evaluation II:4.11%). The procedure involved balloon valve fracture (BVF) using a 22 × 40 mm Atlas Gold PTA Dilatation Catheter, followed by implantation of a 26 mm Medtronic CoreValve Evolut PRO bioprosthesis. The dilatation catheter was connected to a syringe and an indeflator via a high-pressure stopcock. Under rapid pacing at 180 b.p.m., manual balloon inflation was first performed to achieve rapid expansion, after which the stopcock was opened to the indeflator and pressure was gradually increased until fracture of the original bioprosthetic valve ring occurred. Successful fracture was confirmed by the visible release of the balloon waist and separation of the stent posts from the suture cuff under fluoroscopy within 15 s (*Figure 3*; Supplementary material online, *Video S3*). The patient underwent ViV-TAVR in December 2023 successfully without any peri-procedural complications.

At 1-month post-ViV-TAVR, TTE showed improved haemodynamics (pVel: 221.3 cm/s, MPG: 10.3 mmHg), slightly recovered LVGLS (15.5%), and NT-proBNP (1702 pg/mL). However, a follow-up CT in May 2024 revealed HALT in all three aortic cusps, along with thrombus formation in the right sinus of Valsalva, extending into the right coronary artery orifice (*Figure 2B*, top; Supplementary material online, *Video S4*). Despite these findings, TTE demonstrated only mildly elevated pVel (289.7 cm/s; MPG: 11.9 mmHg, EOAi: 0.99 cm^2^/m^2^). Warfarin 1 mg per day was initiated with prothrombin time–international normalized ratio maintained at approximately 1.3. Six months later, follow-up CT confirmed thrombus resolution (*Figure 2B*, bottom), and TTE showed improved haemodynamics (pVel: 226.7 cm/s, MPG: 10.4 mmHg, EOAi: 1.09 cm^2^/m^2^). The patient has since continued warfarin therapy with regular TTE monitoring.

## Discussion

Bioprosthetic valve dysfunction can result from structural or non-structural causes, necessitating multimodality imaging to assess valvular structures and haemodynamic changes.^1,2^ Transthoracic echocardiography serves as the primary imaging modality,^4^ enabling haemodynamic assessment and visualization of valve morphology and mobility. Nevertheless, TTE has limited sensitivity for detecting pannus and valve thrombosis, as both may present with seemingly normal leaflet morphology and motion.^1,2^ In such cases, CT may provide additional diagnostic value, identifying pannus as a hypodense structure near the valve ring and valve thrombosis as HALT or reduced leaflet motion (RLM).^1^

Non-structural BVD, which includes PPM, paravalvular regurgitation, and inappropriate valve positioning, typically manifests immediately after AVR and can be distinguished from structural BVD by comparing follow-up TTEs with the postoperative baseline.^1,4^ However, in our patient, the absence of postoperative baseline TTE made the differentiation particularly challenging. The initial TTE follow-up from 2021 to 2022 showed an acceleration time of <100 ms, without abnormal valve morphology or progression of peak velocity, supporting the diagnosis of PPM. However, the pVel elevation, leaflet thickening, and restricted aortic valve opening from 2022 to 2023 suggested that the coexistence of PPM and BVD may have developed subsequently.

While our patient had no smoking, diabetes, or chronic kidney disease history, he had undergone valve replacement more than a decade earlier.^4^ His receipt of a stented valve and relatively young age (60 years) at implantation were also recognized risk factors for BVD.^4^ Additionally, PPM has been associated with an increased risk of structural BVD.^5^ Current guidelines recommend annual TTE follow-up for surgical bioprosthetic valves beyond 10 years post-implantation.^1^ As in our case, the detection of new-onset valvular degeneration further highlights the importance of individualized follow-up intervals based on patient-specific risk factors.

During ViV-TAVR, we performed BVF before implanting the new bioprosthesis to accommodate a larger valve size, improve frame expansion, and optimize leaflet function and haemodynamics.^6^ Balloon valve fracture is particularly advantageous for patients with smaller surgical aortic valves (≤21 mm) or severe pre-existing PPM, as seen in our case. Currently, no expert consensus exists regarding the timing of BVF—whether before (pre-dilatation) or after ViV-TAVR (post-dilatation).^6^ While post-dilatation carries a lower risk of acute aortic regurgitation and haemodynamic collapse, pre-dilatation minimizes leaflet damage and ensures successful valve fracture.^6^ Unlike native valve TAVR, annular rupture during ViV-TAVR is rare, likely due to the protective effect of the surgical bioprosthetic ring.^7^ Nevertheless, experts have recommended avoiding BVF in cases with heavily calcified aortic roots.^8^

In our patient, the previous Magna Ease valve required relatively high fracturing pressure (18 atm).^6^ Additionally, the waist diameter of the Medtronic Evolut valve and the recommended balloon size for BVF were both 22 mm, increasing the risk of leaflet damage. Given these considerations, we opted for pre-dilatation as the preferred approach.

Valve thrombosis, a frequently observed imaging finding after TAVR, is commonly detected using TTE initially and following confirmation by contrast-enhanced CT. Transthoracic echocardiography remains the first-line screening tool for assessing haemodynamic status, as well as the severity of HALT and RLM.^1^ Previous studies have indicated HALT as a dynamic phenomenon, with spontaneous resolution observed in over 50% of patients within 1 year.^1^ However, the association between post-TAVR HALT or RLM with all-cause mortality or cerebrovascular events remains unclear.^1^ While anticoagulants have demonstrated superior efficacy over dual antiplatelet therapy in preventing or resolving HALT in most cases,^9,10^ rivaroxaban-based therapies have been associated with an increased risk of mortality, thromboembolic, and bleeding events.^10^ Therefore, current guidelines recommend against routinely using anticoagulants after TAVR unless clinically indicated.^4,11^

Notably, previous studies have suggested that ViV-TAVR may carry a higher risk of leaflet thrombosis compared to native TAVR,^3^ which may result from decreased flow and shear stress secondary to flow stasis.^12^ Nevertheless, the number of ViV-TAVR patients included in prior studies has been limited. Given the insufficient data from prior studies, whether guideline-recommended anticoagulation therapy plays a pivotal role in preventing HALT and influences prognosis in patients post–ViV-TAVR remains controversial.

## Conclusion

Bioprosthetic valve dysfunction is a common complication after AVR and requires multimodality and serial imaging to differentiate underlying causes. We also demonstrated the potential effectiveness of warfarin in treating HALT after ViV-TAVR. However, the factors influencing HALT development, including deployment technique, optimal valve expansion, the choice between warfarin and direct oral anticoagulants, and the long-term prognostic impact, warrant further investigation.

## Lead author biography


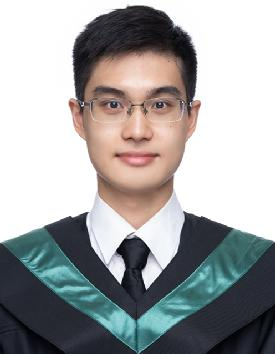


Kang Liu is a post-graduate year resident at the National Taiwan University Hospital. He has an enthusiastic mind for exploring new medical knowledge.

## Supplementary Material

ytag032_Supplementary_Data

## Data Availability

The data underlying this article are available in the article and in its online Supplementary material.
