# Supplementary material for: Valve-in-valve transcatheter aortic valve replacement for bioprosthetic valve failure complicated by hypo-attenuated leaflet thickening: a case report
Source: Eur Heart J Case Rep. 2026 Jan 27;10(2):ytag032. doi: 10.1093/ehjcr/ytag032 (PMC12903473; doi:10.1093/ehjcr/ytag032)
Supplement: ytag032_Supplementary_Data [file ytag032_supplementary_data.zip › Supplemental_Materials.docx]

**Video S1.** **Transthoracic echocardiography at the initial presentation.** The apical four-chamber view showed normal valve morphology, mobility, and absence of leaflet calcification.

**Video S2. Follow-up transthoracic echocardiography.** The apical four-chamber view revealed limited aortic valve opening.

**Video S3**. **Valve-in-valve transcatheter aortic replacement with preprocedural ballon valve fracture.**

**Video S4. Electrocardiography-gated computed tomography after valve-in-valve transcatheter aortic valve replacement.**
